# 503 The Accuracy of the Revised Baux Score in Predicting Burn Mortality: A Systematic Review

**DOI:** 10.1093/jbcr/irad045.100

**Published:** 2023-05-15

**Authors:** Stephanie Bond, Michael Edgar, Sam Jiang, Isabel Scharf, Geronimo Bejarano, Sebastian Vrouwe

**Affiliations:** University of Chicago, Chicago, Illinois; UICOM, Chicago, Illinois; University of Illinois College of Medicine at Chicago, Chicago, Illinois; University of Illinois at Chicago College of Medicine, Chicago, Illinois; University of Texas Health Science Center (UTHealth), Austin, Texas; University of Chicago, Chicago, Illinois; University of Chicago, Chicago, Illinois; UICOM, Chicago, Illinois; University of Illinois College of Medicine at Chicago, Chicago, Illinois; University of Illinois at Chicago College of Medicine, Chicago, Illinois; University of Texas Health Science Center (UTHealth), Austin, Texas; University of Chicago, Chicago, Illinois; University of Chicago, Chicago, Illinois; UICOM, Chicago, Illinois; University of Illinois College of Medicine at Chicago, Chicago, Illinois; University of Illinois at Chicago College of Medicine, Chicago, Illinois; University of Texas Health Science Center (UTHealth), Austin, Texas; University of Chicago, Chicago, Illinois; University of Chicago, Chicago, Illinois; UICOM, Chicago, Illinois; University of Illinois College of Medicine at Chicago, Chicago, Illinois; University of Illinois at Chicago College of Medicine, Chicago, Illinois; University of Texas Health Science Center (UTHealth), Austin, Texas; University of Chicago, Chicago, Illinois; University of Chicago, Chicago, Illinois; UICOM, Chicago, Illinois; University of Illinois College of Medicine at Chicago, Chicago, Illinois; University of Illinois at Chicago College of Medicine, Chicago, Illinois; University of Texas Health Science Center (UTHealth), Austin, Texas; University of Chicago, Chicago, Illinois

## Abstract

**Introduction:**

Osler *et al*. revised the Baux score in 2010 to include inhalation injury, attempting to improve upon the original 1961 model given the significant advances in modern burn care. Other predictive models of burn mortality have similarly been developed using use an assortment of other variables, however there is no consensus on the preferred formula to use. This systematic review aims to investigate the accuracy of the revised Baux (rBaux) score in comparison to other models when determining mortality risk in patients with burn injuries.

**Methods:**

A systematic review was performed by two independent reviewers using electronic databases (PubMed, EMBASE, Scopus) from March 1, 2010 to May 22, 2022. The majority of studies were classified as “high” quality and no studies were excluded due to risk of bias. Articles were included if they were published in English and assessed risk of mortality from burns using the rBaux model and an alternative model. The PRISMA statement was followed, and all the PROBAST quality appraisal checklist was used during data extraction.

**Results:**

The search identified 79 non-duplicate citations, of which 33 underwent full-text review, and 21 met inclusion criteria. They assessed the predictive ability of the rBaux score in comparison to the original Baux score, Belgian Outcome for Burn Injury, Abbreviated Burn Severity Index, APACHE II, Sequential Organ Failure Assessment score, Boston Group/Ryan scores, the FLAMES model, the Prognostic Burn Index, and several other equations that have not been externally validated. The accuracy of mortality prediction (area under ROC curves) for the rBaux score ranged from 0.68 to 0.99 and 14 studies found a value greater than 0.90. These ROC values were found to be lowest in the two studies with subgroupings for age, demonstrating an ROC of 0.01 in children, and 0.68 and 0.92 in the elderly. Eleven studies described sensitivity, specificity, or both, with sensitivity ranging from 12% to 96%, and specificity ranging from 80% to 100%.

**Conclusions:**

The rBaux score offers a relatively easy method to rapidly assess the mortality risk from burn injury in a broad range of patient populations based on age, percent total body surface area burned, and presence/absence of an inhalation injury. This tool remains valid despite the exclusion of patient comorbidities and other injury-related factors. However, this study also identified the rBaux’s ability to predict mortality risk was limited when applied to patients at the extremes of age, highlighting an important area for future research. At present, given its relative simplicity and generalizability, the rBaux score remains a useful clinical tool for predicting mortality.

**Applicability of Research to Practice:**

The rBaux score remains a simple yet powerful predictor of mortality in burn patients, which can be applied at the bedside on initial presentation across diverse patient populations.

